# The Relationship Between Sleep Duration and Blood Lipids Among Chinese Middle-Aged and Older Adults: Cross-Lagged Path Analysis From CHARLS

**DOI:** 10.3389/fpubh.2022.868059

**Published:** 2022-05-13

**Authors:** Ziwei Chen, Xia Zhang, Yanran Duan, Tingting Mo, Wenli Liu, Yilei Ma, Ping Yin

**Affiliations:** ^1^Department of Epidemiology and Biostatistics, School of Public Health, Tongji Medical College, Huazhong University of Science and Technology, Wuhan, China; ^2^The First People's Hospital of Jingzhou, Jingzhou, China; ^3^Department of Occupational and Environmental Health, School of Public Health, Tongji Medical College, Huazhong University of Science and Technology, Wuhan, China; ^4^Department of Statistics, East China Normal University, Shanghai, China

**Keywords:** sleep duration, blood lipids, cross-lagged panel model, temporal relationship, Chinese middle-aged and elderly individuals

## Abstract

This study used data from the China Health and Retirement Longitudinal Study to investigate the temporal relationship between blood lipids and sleep duration in Chinese middle-aged and older adults. We used medical examinations and questionnaire data of 5,016 Chinese middle-aged and older adults (age 45+) in 2011 and 2015. Cross-lagged path analysis was performed to examine the bidirectional relationships between blood lipids and sleep duration. Sleep duration and lipids data were analyzed as continuous variables. Temporal relationships between sleep duration and HDL-cholesterol, LDL-cholesterol, total cholesterol, and triglycerides were different. Sleep duration was negatively associated with HDL-cholesterol 4 year later (β_1_ = −0.171, *P* = 0.005), and HDL-cholesterol was negatively associated with sleep duration 4 year later (β_2_ = −0.006, *P* = 0.002). Longer sleep duration was associated lower levels of LDL-cholesterol (β_1_ = −0.275, *P* = 0.097) and total cholesterol (β_1_ = −0.329, *P* = 0.096) 4 year later. There was a positive correlation between triglycerides and sleep duration. The path coefficient from triglycerides to sleep duration 4 year later (β_2_ = 0.001, *P* = 0.018) was greater than that from sleep duration to triglycerides 4 year later (β_1_ = 0.109, *P* = 0.847), with *P* = 0.030 for the difference between β_1_ and β_2_. In stratified analysis, we found that the strength and direction of the relationships may be related to age and BMI. Effects of sleep duration on blood lipids were only observed among participants aged <60 years, while the effect in the opposite direction was observed in older adults (age 60+), and the cross-lagged path coefficients were more significant in adults with BMI > 25.

## Introduction

A series of epidemiological and observational studies have shown that both long and short sleep duration are associated with hypertension, diabetes, stroke, and coronary heart disease mortality (1–5). Research has repeatedly documented that dyslipidemia is a major risk factor of cardiovascular-related diseases and other chronic epidemics, especially for middle-aged and older adults, and the number of people with dyslipidemia steadily increased over the past few years (6–8). As the essential role of sleep in metabolic homeostasis is increasingly recognized, a growing number of cross-sectional studies have investigated significant associations between self-reported sleep variables and lipids (9–11). One cross-sectional study reported that the odds for meeting the elevated triglyceride criterion (triglycerides ≥ 150 mg/dL or use of dyslipidemic medication) were increased by 53% in short sleepers, compared with the reference group of men and women who slept 7–8 h per night (11). Prospective data have indicated the impact of sleep duration on lipids, metabolic syndrome and other risk factors of cardiovascular diseases, but some of these results were inconsistent, possibly due to different study designs and heterogeneity of the study population (12–17). Chinese middle-aged and older adults are the high-risk group of sleep disorders and dyslipidemia and have different physical conditions and living habits from the Western population (18). To date, there have been no studies focusing on the association between sleep duration and blood lipids in Chinese middle-aged and older adults.

In addition, it remains unclear whether sleep is a cause, a result, or merely a symptom of diseases such as dyslipidemia, which is critical to understand the mechanism behind the detected associations. Most of the existent longitudinal research has typically made an implicit assumption that the flow of causation is in one direction, i.e., sleep duration may affect triglyceride level, but not vice versa (14). However, this assumption is not necessarily reasonable, and it is crucial to know which comes first for the primary and secondary prevention of disease (19, 20). There are three possible directions of effect between sleep duration and blood lipid levels: (i) sleep duration is causally related to blood lipid levels, (ii) blood lipid levels are causally related to sleep duration, and (iii) the direction of causal effects flows both ways. There is a lack of evidence in prospective studies indicating the effect of blood lipid levels on sleep duration, and few researchers have investigated the reciprocal relationship between sleep duration and blood lipid levels. Therefore, we aimed to examine the directionality between sleep duration and blood lipid levels using a cross-lagged technique with a two-wave design.

We focused on Chinese middle-aged and older adults, and used data from a large nationally representative longitudinal cohort to assess temporal relationships between sleep duration and blood lipid levels, especially the possible bidirectional relationships. We also tested whether these associations varied by age and body mass index because sleep duration and blood lipid levels varied among these groups.

## Methods

### Study Population and Survey Methods

Data for this present prospective study was derived from the China Health and Retirement Longitudinal Study (CHARLS), which was an ongoing cohort study of middle-aged and older adults in China conducted by the National School for Development at Peking University. The multistage stratified probability sampling technique was used in CHARLS to get a representative population sample obtained from 150 counties within 28 provinces, municipal cities, and autonomous regions. The CHARLS collected information on demographics and household information, biomedical measurement, health status, and functioning, etc. with face-to-face computer-assisted personal interviews (CAPIs) for the first time in 2011–2012 and every 2 years thereafter. The CHARLS was approved by the Ethics Committee of Peking University Health Science Center. Details of the survey design have been described elsewhere (21).

**Figure 1:**
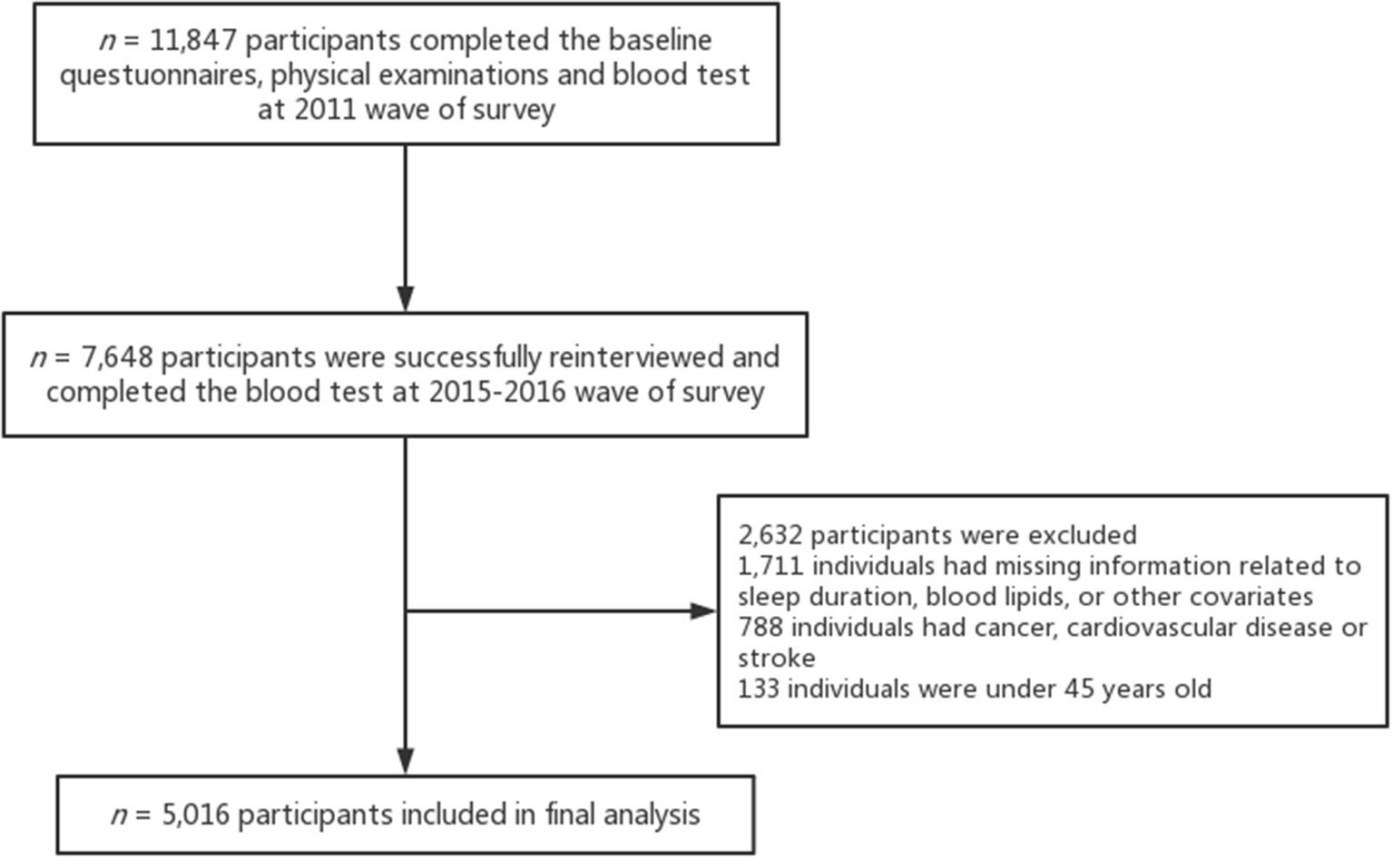
The flowchart of the inclusion of participants.

The baseline questionnaires and physical examinations were conducted with 17,705 participants and 11,847 respondents (67%) completed the blood test. Of these, 10,384 participants were successfully re-interviewed in 2015–2016 and 7,648 individuals (74%) of them completed the blood test. Additionally, we excluded 1,711 individuals with missing information related to sleep duration, blood lipids, or other covariates. The final sample was composed of 5,016 adults after excluding individuals with cancer, cardiovascular disease, or stroke (*n* = 788), and those who were under 45 years old (*n* = 133). See Figure 1 for further details on the inclusion and exclusion of study participants.

### Study Variables

Nightly duration of sleep was assessed by asking subjects the following questions, “During the past month, how many hours of actual sleep did you get at night (average hours for one night)? (This may be shorter than the number of hours you spend in bed.).” Afternoon naps were assessed by asking, “During the past month, how long did you take a nap after lunch in general?” Participants were classified as either having regular lunchtime naps or not. Triglycerides (TG), high-density lipoprotein cholesterol (HDL-C), low-density lipoprotein cholesterol (LDL-C), and total cholesterol (TC) concentrations were determined using an enzymatic colorimetric test. All respondents were asked to have fasted overnight. Sleep duration and lipids data were analyzed as continuous variables in the cross-lagged analysis.

### Covariates

Sociodemographic characteristics, habits and customs, and health status were evaluated as potential confounders of relationships between sleep duration and blood lipids. Age, gender, region (rural-urban), educational level, medical insurance, and marital status were obtained through a self-reported questionnaire at the baseline survey. The health-related covariates included smoking and alcohol consumption. Participants who had either completely quit smoking or had never smoked were defined as non-current smokers. Current drinkers were defined as if they drank liquor, beer, wine, or other alcoholic beverage more than once a month in the last year. The depressive symptoms of the participants were evaluated using the 10-item Center for Epidemiologic Studies Depression Scale (CESD-10) short form. Each item was scored on a 4-point scale, with a total possible score ranging from 0 to 30, and the score of 12 was used as a cutoff point to determine binary depression symptoms (22). Diastolic and systolic pressures were the averages of the three repeated measurements of blood pressures, which were measured by an Omron (Dalian, China) HEM-7200 monitor after the participants had rested for 30 min. Bodyweight, height and circumference were measured to the nearest 0.1 kg, 0.1 cm, and 0.1 cm, respectively, with subjects in light indoor clothing. Abdominal obesity was defined as a waist circumference ≥90 in man and ≥80 in women (23). The venous blood samples were collected during the investigation by trained nurses and were transported based on standard protocol to the Chinese Center for Disease Control and Prevention within 2 weeks, where the samples were stored at −80 and then were assessed at the Clinical Laboratory of Capital Medical University. The glucose was measured using the enzymatic colorimetric test, and HbA1c was measured using boronate affinity chromatography (24).

### Statistical Analysis

**Figure 2:**
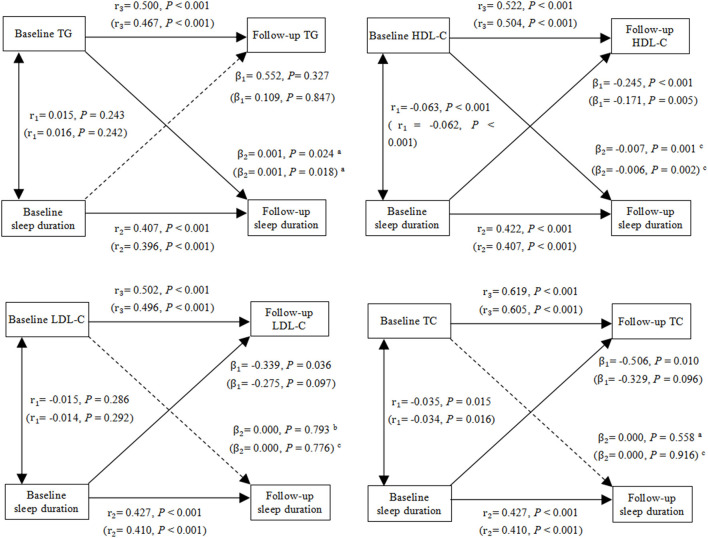
Cross-lagged path analysis of TG, HDL-C, LDL-C, TC and sleep duration. The results outside brackets came from initial models, and the results in brackets came from adjusted models. Initial model was adjusted for no covariates. Adjusted model was adjusted for gender, BMI, age, smoking status, drinking status, region, marital status, education levels, medical insurance, systolic, diastolic, glucose and HbA1c. *r*_1_, synchronous correlation coefficients; *r*_2_ and *r*_3_, stability paths coefficients; β_1_ and β_2_, cross-lagged path coefficients. Goodness-of-fit (initial model/adjusted model): TG: CFI = 1.000/1.000, SRMR = 0.003/0.001, RMSEA = 0.000/0.014; HDL-C: CFI = 0.997/0.998, SRMR = 0.011/0.002, RMSEA = 0.047/0.038; LDL-C: CFI = 1.000/1.000, SRMR = 0.005/0.001, RMSEA = 0.017/0.004; TC: CFI = 0.999/1.000, SRMR = 0.006/0.001, RMSEA = 0.025/0.000. ^a^*P* < 0.05 for the statistically significant difference between β_1_ and β_2_. ^b^0.05 ≤ *P* ≤ 0.1 for the marginal statistically significant difference between β_1_ and β_2_. ^c^*P* > 0.1 for the non-statistically significant difference between β_1_ and β_2_.

Continuous and categorical variables are presented as the mean ± SEM or as numbers and percentages, respectively. The significance of differences between BMI and age groups was assessed by non-parametric tests for skewed continuous variables, and χ^2^ tests for categorical variables. As a form of path analysis, cross-lagged panel model was used to estimate the directional influence inter-related variables have on each other over time in the field of sociology and medicine (25–27). Figure 2 provides graphical representations of the cross-lagged panel model. Cross-lagged panel model is a kind of structural equation modeling, which can examine the causal relationship between variables. Instead of modeling separately, this approach can simultaneously detect the relationship between two variables over 2 years by testing stability paths (e.g., sleep duration at baseline to sleep duration at follow-up), concurrent paths (e.g., sleep duration at baseline and HDL-C at baseline), and cross-lagged paths (e.g., sleep duration at baseline to HDL-C at follow-up; HDL-C at baseline to sleep duration at follow-up). According to the principle that the paths in a model cannot be saturated, the residual terms of the two dependent variables (e.g., sleep duration at follow-up and HDL-C at follow-up) were assumed to be uncorrelated. The robust maximum likelihood method, which was recommended to produce efficient estimates of the parameters in the structural equation model when the observations did not satisfy the assumption of normal distribution, was used to estimate the parameters in the cross-lagged panel model (28). The model fit was evaluated using the comparative fit index (CFI) (≥ 0.90 was considered acceptable) (29), the Standardized Root Mean Square Residual (SRMR) (<0.08 was considered acceptable) (30), and Root Mean Square Error of Approximation (RMSEA) (<0.06 was considered acceptable) (30). The difference between β_1_ and β_2_ derived from the standardized variables (Z-scores) was examined using Fisher's Z test. Stratified analyses were performed by baseline characteristics (age [45- <60, ≥ 60 years], BMI [ ≤ 25, > 25 kg/*m*^2^] to test the difference in cross-lagged path parameters between groups. Population was divided into under-weight (BMI <18.5 kg/*m*^2^), normal weight (18.5 ≤ BMI <25 kg/*m*^2^), overweight (25 ≤ BMI <30 kg/*m*^2^) and obese (BMI ≥ 30 kg/*m*^2^) according to World Health Organization divided population. We set the BMI cut-off point to 25, as participants with a BMI below 18.5 or >30 accounted for only about 8% of the total. The cut-off point for age was set at 60 years, giving rise to a middle-aged and older adults group. The initial model was used to test the potential bidirectional associations between sleep duration and blood lipids, including triglyceride, HDL-C, LDL-C and TC. Potential confounders were adjusted for gender, smoking status, drinking status, region, marital status, education, insurance status, depression, systolic, diastolic, glucose, HbA1c, BMI (not included in the stratified analysis according to BMI) and age (not included in the stratified analysis according to age). The path analyses were conducted in R by using package *lavaan*, which had been developed to provide applied researchers package for structural equation modeling (31). All the statistical analyses were performed using R version 3.5.3 (R Foundation for Statistical Computing, Vienna, Austria).

## Results

### Descriptive Analysis

Table 1 provides a descriptive summary of the variables for the entire study population categorized by BMI and age group. Nearly 85% of all participants lived in rural areas, and more than 73% of the respondents were non-illiterate. Sleep duration at baseline and follow-up differed at statistical significance among the BMI and age categories, and the proportion of participants who sleep longer (>9 h) or shorter (<6 h) was higher in the older people. Among subjects with BMI > 25 *kg/m*^2^, LDL-C, TC and TG were significantly higher, and HDL-C was significantly lower compared with those with lower BMI. By contrast, only baseline blood lipid levels were significantly or marginally different between age groups. Participants over 60 years old tended to have higher glucose, HbA1c and pulse pressure. Pulse pressure was defined as the difference between systolic and diastolic measurements of blood pressure. And those with higher BMI tended to have higher systolic, diastolic blood pressure, glucose and HbA1c.

**Table 1:** Descriptive data of study variables by baseline age and BMI (China Health and Retirement Longitudinal Study 2011–15).

**Variable**	**Overall (*N* = 5,016)**	**Age group**			**BMI group**		
		**45- <60 years**	**≥ 60 years**	***P*-value^a^**	**≤25 *kg/*m^2^**	**> 25 *kg/*m^2^**	***P*-value^b^**
		**(*****N*** **=** **2,671)**	**(*****N*** **=** **2,345)**		**(*****N*** **=** **3,492)**	**(*****N*** **=** **1,524)**	
Sleep duration at baseline (h)				<0.001			<0.001
<6	1,419 (28.29)	642 (24.04)	777 (33.13)		1,051 (30.10)	368 (24.15)	
6- <9	3,190 (63.60)	1,824 (68.29)	1,366 (58.25)		2,149 (61.54)	1,041 (68.31)	
≥9	407 (8.11)	205 (7.68)	202 (8.61)		292 (8.36)	115 (7.54)	
Sleep duration at follow-up (h)				<0.001			<0.001
<6	1,555 (31.00)	775 (29.02)	780 (33.26)		1,105 (31.64)	450 (29.53)	
6- <9	2,971 (59.23)	1,680 (62.89)	1,291 (55.05)		2,040 (58.42)	931 (61.09)	
≥9	490 (9.77)	216 (8.09)	274 (11.69)		347 (9.93)	143 (9.38)	
Blood lipids at baseline (mg/dl)							
TG	130.24 ± 94.52	135.13 ± 102.57	124.66 ± 84.11	0.002	117.39 ± 80.76	159.67 ± 115.00	<0.001
HDL-C	51.53 ± 15.40	50.79 ± 14.95	52.38 ± 15.86	<0.001	54.00 ± 15.66	45.87 ± 13.15	<0.001
LDL-C	116.32 ± 34.43	115.09 ± 33.97	117.71 ± 34.91	0.007	114.88 ± 33.53	119.61 ± 36.22	<0.001
TC	193.39 ± 37.42	192.45 ± 36.77	194.47 ± 38.14	0.066	191.63 ± 37.02	197.43 ± 38.05	<0.001
Blood lipids at follow-up (mg/dl)							
TG	141.91 ± 89.41	148.42 ± 93.87	134.48 ± 83.45	<0.001	129.43 ± 81.81	170.50 ± 99.03	<0.001
HDL-C	51.85 ± 12.06	51.63 ± 11.78	52.11 ± 12.36	0.269	53.39 ± 12.66	48.35 ± 9.66	<0.001
LDL-C	103.23 ± 28.60	102.97 ± 28.86	103.52 ± 28.30	0.333	102.37 ± 28.25	105.18 ± 29.31	0.002
TC	185.55 ± 36.39	186.00 ± 36.75	185.05 ± 35.99	0.504	184.15 ± 36.43	188.77 ± 36.12	<0.001
Male gender (%)	2,368 (47.21)	1,143 (42.80)	1,225 (52.24)	<0.001	1,803 (51.63)	565 (37.07)	<0.001
Age (years)	59.31 ± 8.54	52.78 ± 4.19	66.76 ± 5.64	<0.001	60.09 ± 8.76	57.53 ± 7.72	<0.001
BMI (*kg/m*^2^)	23.52 ± 3.75	24.03 ± 3.76	22.95 ± 3.66	<0.001	21.64 ± 2.09	27.85 ± 3.06	<0.001
Regular lunchtime naps (%)	2,105 (41.97)	1,107 (41.45)	998 (42.56)	0.442	1,443 (41.32)	662 (43.44)	0.172
Current smoking (%)	1,570 (31.30)	786 (29.43)	784 (33.43)	0.002	1,249 (35.77)	321 (21.06)	<0.001
Current drinking (%)	1,730 (34.49)	916 (34.29)	814 (34.71)	0.779	1,297 (37.14)	433 (28.41)	<0.001
Region (%)				0.011			<0.001
Urban	745 (14.86)	429 (16.06)	316 (13.48)		445 (12.74)	300 (19.69)	
Rural	4,271 (85.15)	2,242 (83.94)	2,029 (86.52)		3,047 (87.26)	1,224 (80.31)	
Marital status (%)				<0.001			<0.001
Single	672 (13.40)	177 (6.63)	495 (21.11)		524 (15.01)	148 (9.71)	
Married or cohabiting	4,344 (86.60)	2,494 (93.37)	1,850 (78.89)		2,968 (84.99)	1,376 (90.29)	
Education (%)				<0.001			<0.001
Illiterate	1,340 (26.71)	536 (20.07)	804 (34.29)		949 (27.18)	391 (25.66)	
Primary school	2,148 (42.82)	1,000 (37.44)	1,148 (48.96)		1,553 (44.47)	595 (39.04)	
Middle school or beyond	1528 (30.47)	1135 (42.49)	393 (16.76)		990 (28.35)	538 (35.30)	
With medical insurance (%)	4,763 (94.96)	2,535 (94.91)	2,228 (95.01)	0.920	3,319 (95.05)	1,444 (94.75)	0.712
Depression (%)	1,081 (21.55)	523 (19.58)	558 (23.80)	<0.001	796 (22.79)	285 (18.70)	0.001
Systolic (mmHg)	131.63 ± 22.03	128.05 ± 20.34	135.70 ± 23.15	<0.001	129.30 ± 21.54	136.96 ± 22.20	<0.001
Diastolic (mmHg)	76.54 ±12.71	77.22 ± 13.18	75.78 ± 12.11	0.001	74.79 ±12.30	80.56 ± 12.73	<0.001
Glucose (mg/dl)	109.13 ± 33.97	107.22 ± 32.25	111.31 ± 35.72	<0.001	107.48 ± 32.57	112.91 ± 36.72	<0.001
HbA1c (mg/dl)	5.25 ± 0.77	5.21 ± 0.77	5.30 ± 0.78	<0.001	5.20 ± 0.73	5.37 ± 0.85	<0.001

*Values are mean ± SD or n (percentage).*

*TG, Triglycerides; HDL-C, High-density lipoprotein cholesterol; LDL-C, Low-density lipoprotein cholesterol; TC, Total cholesterol; BMI, Body mass index.*

a *P-values for differences between age categories.*

b *P-values for differences between BMI categories*.

To examine whether there are linear relationships between sleep duration and blood lipid levels 4 years later as well as blood lipid levels and sleep duration 4 years later, Table 2 presents the means and confidence intervals for TG, HDL-C, LDL-C, and TC in the different sleep duration groups. TG and sleep duration at baseline were positively correlated with sleep duration and TG at follow-up, respectively. The tracking correlations of HDL-C and sleep duration from baseline to follow-up and the synchronous correlations between HDL-C and sleep duration at baseline and follow-up were both negative. The results for LDL-C and TC were similar to those of HDL-C, showing negative tracking correlations between blood lipid levels and sleep duration. The directions of correlations among the study variables showed consistency with the proposed cross-lagged models.

**Table 2:** Mean values and 95% confidence intervals of TG, HDL-C, LDL-C and TC in different sleep duration groups.

	**Baseline sleep duration (h)**			**Follow-up sleep duration (h)**		
	** <6**	**6–9**	**≥9**	** <6**	**6–9**	**≥9**
Blood lipid levels at baseline (mg/dl)						
TG	127.91 (122.79, 133.03)	129.93 (126.63, 133.22)	137.36 (129.54, 145.18)	126.10 (121.15, 131.06)	130.25 (126.89, 133.62)	137.56 (130.11, 145.01)
HDL-C	52.69 (51.80, 53.59)	51.41 (50.88, 51.93)	49.72 (48.46, 50.98)	52.88 (52.00, 53.76)	51.37 (50.83, 51.91)	49.85 (48.71, 51.0)
LDL-C	116.03 (114.09, 117.98)	116.69 (115.52, 117.87)	114.56 (111.54, 117.58)	117.49 (115.46, 119.53)	116.06 (114.90, 117.23)	115.39 (112.64, 118.13)
TC	194.35 (192.23, 196.47)	193.33 (192.06, 194.61)	191.65 (188.36, 194.93)	195.33 (193.16, 197.49)	192.84 (191.56, 194.12)	192.52 (189.51, 195.53)
Blood lipid levels at follow-up (mg/dl)						
TG	140.01 (134.68, 145.33)	141.72 (138.73, 144.71)	147.31 (139.47, 155.15)	138.34 (133.43, 143.25)	142.75 (139.58, 145.92)	144.36 (137.70, 151.03)
HDL-C	52.77 (52.07, 53.47)	51.86 (51.45, 52.28)	49.76 (48.78, 50.74)	53.39 (52.71, 54.06)	51.65 (51.22, 52.07)	50.08 (49.19, 50.97)
LDL-C	103.69 (102.06, 105.32)	103.43 (102.45, 104.41)	100.88 (98.48, 103.28)	105.73 (104.06, 107.40)	102.54 (101.56, 103.53)	101.92 (99.74, 104.09)
TC	186.99 (184.84, 189.13)	185.53 (184.31, 186.75)	182.51 (179.30, 185.72)	189.47 (187.28, 191.66)	184.55 (183.32, 185.77)	183.21 (180.33, 186.09)

### Cross-Lagged Path Analysis of Sleep Duration and Blood Lipids

Figure 2 shows cross-lagged path analysis of sleep duration and blood lipid levels in the total population. After adjusting for gender, age, smoking status, drinking status, region, marital status, education, medical insurance, depression, systolic, diastolic glucose, and glycosylated hemoglobin, there was a significant bidirectional relationship between sleep duration and HDL-C level. Sleep duration was negatively associated with HDL-C 4 year later (β_1_ = −0.171, *P* = 0.005), and HDL-C was negatively associated with sleep duration 4 year later (β_2_ = −0.006, *P* = 0.002). In contrast, there was a positive correlation between TG and sleep duration, and the path coefficient from TG to sleep duration 4 year later (β_2_ = 0.001, *P* = 0.018) was greater than the path coefficient from sleep duration to TG 4 year later (β_1_ = 0.109, *P* = 0.847), with *P* = 0.030 for the difference between β_1_ and β_2_. Moreover, longer sleep duration were significantly associated lower levels of LDL-C (β_1_ = −0.339, *P* = 0.036) and TC (β_1_ = −0.506, *P* = 0.010) 4 year later, and the sleep duration were marginally significantly associated with LDL-C (β_1_ = −0.275, *P* = 0.097) and TC (β_1_ = −0.329, *P* = 0.096) 4 year later after adjusting for covariates. Fit indexes of all the cross-lagged models indicated a good fit to the observed data according to the criteria of CFI ≥ 0.90, SRMR <0.08, and RMSEA <0.06.

### Cross-Lagged Path Analyses by Subgroups

Tables 3, 4 provide a summary of cross-lagged path analysis of TG, HDL-C, LDL-C, TC, and sleep duration by age and BMI. Significant longitudinal association between TG at baseline and sleep duration at follow-up was observed among subjects with BMI > 25 *kg/m*^2^ (β_2_ = 0.001, *P* = 0.019) or age ≥ 60 years old (β_2_ = 0.001, *P* = 0.026). The direction of effect between HDL-C and sleep duration which was bidirectional in all subjects differed across various subgroups. HDL-C at baseline was significantly associated with sleep duration at follow-up subgroups of BMI > 25 *kg/m*^2^ (β_2_ = −0.012, *P* = 0.001) or age ≥ 60 years old (β_2_ = −0.011, *P* = 0.001) with *P* = 0.003 and *P* = 0.046 for the difference in the path coefficients (β_1_ and β_2_), respectively. In contrast, the path coefficients from sleep duration at baseline to HDL-C at follow-up in subgroups of BMI ≤ 25 *kg/m*^2^ (β_1_ = −0.267, *P* = 0.001) and aged <60 years (β_1_ = −0.236, *P* = 0.007) were significant. In addition, HDL-C and TG were strongly correlated with BMI in the total study population. At baseline, the correlation coefficient between HDL-C and BMI was −0.284 (*P* < 0.001), and that between TG and BMI was 0.217 (*P* < 0.001).

**Table 3:** Cross-lagged path analysis of TG, HDL-C, LDL-C, TC and sleep duration by age.

		**Path coefficients**					**Goodness-of-fit indices**		
		**Baseline sleep duration to**		**Baseline blood lipids to**		***P*-value ^a^**	**CFI**	**SRMR**	**RMSEA**
		**follow-up blood lipids**		**follow-up sleep** **duration**					
		**β**_**1**_	***P*** **-value**	**β**_**2**_	***P*** **-value**				
Age <60 years (*N* = 2,671)
TG	Initial model	1.483	0.104	0.001	0.078	0.893	0.999	0.007	0.017
	Adjusted model	1.056	0.248	0.000	0.183	0.861	0.999	0.002	0.019
HDL-C	Initial model	−0.284	0.001	−0.003	0.164	0.063	0.999	0.006	0.023
	Adjusted model	−0.236	0.007	−0.002	0.383	0.071	0.999	0.002	0.020
LDL-C	Initial model	−0.440	0.065	−0.001	0.608	0.183	1.000	0.001	0.000
	Adjusted model	−0.436	0.068	0.000	0.984	0.071	1.000	0.000	0.000
TC	Initial model	−0.598	0.049	0.000	0.953	0.056	1.000	0.001	0.000
	Adjusted model	−0.495	0.101	0.000	0.917	0.124	1.000	0.001	0.000
Age ≥ 60 years (*N* = 2,345)
TG	Initial model	−0.518	0.449	0.001	0.110	0.400	1.000	0.000	0.000
	Adjusted model	−0.506	0.465	0.001	0.026	0.134	1.000	0.001	0.000
HDL-C	Initial model	−0.248	0.004	−0.011	<0.001	0.477	0.996	0.013	0.059
	Adjusted model	−0.124	0.152	−0.011	0.001	0.046	0.996	0.003	0.047
LDL-C	Initial model	−0.354	0.112	0.000	0.816	0.175	0.998	0.010	0.038
	Adjusted model	−0.189	0.417	0.001	0.676	0.694	0.999	0.002	0.023
TC	Initial model	−0.596	0.021	−0.001	0.337	0.179	0.996	0.012	0.053
	Adjusted model	−0.259	0.327	0.000	0.920	0.379	0.999	0.002	0.032

*Initial model was adjusted for no covariates.*

*Adjusted model was adjusted for gender, BMI, smoking status, drinking status, region, marital status, education levels, medical insurance, systolic, diastolic, glucose and HbA1c.*

a *P-values derived from Fisher's test for the differences between β_1_ and β_2_*.

**Table 4:** Cross-lagged path analysis of TG, HDL-C, LDL-C, TC and sleep duration by BMI.

		**Path coefficients**					**Goodness-of-fit indices**		
		**Baseline sleep duration to**		**Baseline blood lipids to**		***P*-value ^a^**	**CFI**	**SRMR**	**RMSEA**
		**follow-up blood lipids**		**follow-up sleep duration**					
		**β**_**1**_	***P*** **-value**	**β**_**2**_	***P*** **-value**				
BMI ≤ 25 *kg/m*^2^ (*N* = 3,492)
TG	Initial model	0.410	0.509	0.000	0.420	0.883	0.999	0.007	0.023
	Adjusted model	0.202	0.746	0.000	0.323	0.506	0.998	0.002	0.034
HDL-C	Initial model	−0.305	<0.001	−0.005	0.039	0.055	0.996	0.012	0.052
	Adjusted model	−0.267	0.001	−0.004	0.062	0.114	0.998	0.002	0.036
LDL-C	Initial model	−0.106	0.576	0.000	0.994	0.518	0.999	0.008	0.029
	Adjusted model	−0.036	0.850	0.001	0.445	0.565	1.000	0.001	0.018
TC	Initial model	−0.329	0.161	0.000	0.622	0.363	0.999	0.007	0.029
	Adjusted model	−0.152	0.514	0.000	0.772	0.717	1.000	0.001	0.000
BMI > 25 *kg/m*^2^ (*N* = 1,524)
TG	Initial model	−0.062	0.959	0.001	0.015	0.017	1.000	0.004	0.000
	Adjusted model	−0.321	0.796	0.001	0.019	0.037	1.000	0.001	0.000
HDL-C	Initial model	−0.108	0.241	−0.013	0.001	0.028	1.000	0.007	0.015
	Adjusted model	−0.016	0.863	−0.012	0.001	0.003	0.998	0.003	0.043
LDL-C	Initial model	−1.014	0.001	−0.001	0.386	0.017	1.000	0.002	0.000
	Adjusted model	−0.951	0.004	−0.001	0.365	0.046	1.000	0.001	0.000
TC	Initial model	−1.096	0.002	−0.001	0.609	0.012	1.000	0.003	0.000
	Adjusted model	−0.870	0.020	−0.001	0.487	0.105	1.000	0.000	0.000

*Initial model was adjusted for no covariates.*

*Adjusted model was adjusted for gender, age, smoking status, drinking status, region, marital status, education levels, medical insurance, systolic, diastolic, glucose and HbA1c.*

a *P-values derived from Fisher's test for the differences between β_1_ and β_2_*.

## Discussion

Based on a two-wave longitudinal research design, this study examined the time relationships between sleep duration and blood lipids in a large sample of Chinese middle-aged and older adults. To the best of our knowledge, this study is the first to investigate the temporal relationships between blood lipids and sleep duration using a cross-lagged path analysis model, a statistical method for analyzing the causal relationship between interrelated variables. Some epidemiologic studies reported a U-shaped association between sleep duration and lipids, metabolic syndrome and obesity (16, 32, 33). However, no U-shaped relationship has been found in the study in which demographic characteristics of participants were consistent with ours, since the average level and distribution of blood lipids in Chinese middle-aged and older adults are significantly different from those in other countries or regions (15). Thus, considering the trend of changes in blood lipid levels with sleep duration, the two study variables were included as continuous variables in the path analysis which implied the assumption that the two variables were linearly correlated. Our findings showed that negative associations existed between HDL-C and sleep duration and the relationships were bidirectional across the general population. One other striking observation from the present study was that higher TG preceded longer sleep duration rather than vice versa, which was inconsistent with the natural hypotheses of research focused on the effect of sleep on coronary heart disease. Furthermore, the results of the stratified analysis suggested that the longitudinal association between HDL-C at baseline and sleep duration at follow-up was more pronounced in individuals with BMI > 25 or age ≥ 60 years, while the association in the opposite direction was only observed in the other groups. Similarly, the significant effect of TG on sleep duration was not observed in individuals with BMI ≤ 25 or age <60 years. The findings would provide additional perspective for research on mechanisms linking sleep duration with cardiovascular risk and disease prevention in middle-aged and older adults.

Previous studies have reported some inconsistent findings on the longitudinal effect of sleep duration on HDL-C and a few of these are in concert with our findings (10, 12, 14, 15, 34). The studies that are consistent with our results have focused on the middle-aged and older adults in Asia, whose demographic characteristics were reasonably the same as ours (10, 12, 14). The Dongfeng-Tongji cohort, which is a Chinese longitudinal cohort of middle-aged and older adults, showed that longer sleep duration (≥10 h) was significantly associated with a reduction in HDL-C (14). The finding of short sleep duration (<6 h) was a protective factor for reduced HDL-C was found in two prospective studies which were the Korean Genome and Epidemiology Study of participants aged between 40 and 70 years old and the Guangzhou Biobank Cohort Study of older Chinese (10, 12). However, some other findings were contrary to ours or didn't determine a statistically significant association between sleep duration and HDL-C, which may be due to differences in demographic characteristics of the study population or classification strategies of sleep duration (35, 36). In addition to the findings consistent with other studies, our study also examined the effect of HDL-C on later sleep duration using the cross-lagged model without an assumption about the chronological order of events. To the best of our knowledge, this study is the first to report the bidirectional relationships between HDL-C and sleep duration. It is worth noting the cross-sectional and longitudinal associations reported between sleep duration and HDL-C in previous studies are in large part explained by the demonstrated bidirectional relationships in our studies.

Several epidemiology studies have found a positive correlation between sleep duration and triglyceride level, and prospective studies attempted to demonstrate that long sleep duration is a risk factor for high triglyceride (10, 14, 34). For example, a study from Chicago reported that each hour increase in sleep duration was significantly associated with higher TG(1.1 mg/dL, 95%CI: 1.0, 1.1) among men. However, we found that higher triglyceride level could precede a longer sleep duration. The pathophysiological mechanism mediating the effect of triglyceride on sleep duration remains unclear as few studies explored the longitudinal effect of blood lipids on sleep, but there are some genetic evidence on the mechanisms that elevated triglyceride level is a biomarker of cardiovascular risk (37). Considering longer sleep duration is associated with a higher risk of coronary heart disease incidence, which has been demonstrated in recent prospective studies (14), we speculate that the potential causal pathways are (1) dyslipidemia as well as preexisting cardiovascular morbidity strongly affecting sleeping patterns, (2) sleep partially mediates the association between dyslipidemia and cardiovascular disease or (3) some confounding factors such as risky lifestyles affect the sleep pattern, lipids metabolism and other biomarker's expression levels, resulting in spurious associations between variables. There are several studies that focused on the longitudinal relationship between sleep duration and cardiometabolic risk factors inferring that the effect of sleep on incident coronary heart disease might be mediated by raised TG, decreased HDL-C and so on (14, 38, 39). In contrast, the difference between the two path coefficients in our study suggested a piece of evidence for the temporal relationship between raised triglycerides and long sleep duration, which indicated that raised triglycerides may predict later longer sleep. Additionally, the strength of the cross-lagged paths also demonstrates that HDL-C might be a predictor of later sleep duration. Based on the above findings, we could speculate that the sleep pattern was more likely to be a consequence or a symptom compared with a risk factor for dyslipidemia in certain demographic groups.

Interestingly, we found that the directions and strengths of associations between sleep duration and blood lipids were not consistent in different age and BMI groups. After adjusting for covariates, the effects of sleep duration on blood lipids were only observed among individuals aged <60 years, while the effect in the opposite direction was only observed in older adults. Even HDL-C, which had a bidirectional association with sleep duration in the general population, followed this pattern. Older adults are more likely to develop or exacerbate dyslipidemia due to metabolic disorders caused by other diseases such as hypothyroidism, diabetes and hypertension (40, 41), and they are more likely to experience several sleep disorders, such as more frequent awakening and slow-wave sleep reduction, leading to the weakening of the effect of sleep duration on blood lipids (42, 43). Although the biological mechanisms behind the effect of aging on sleep-lipids associations are not clear yet, from the perspective of disease prevention, relatively healthy middle-aged people are recommended to ensure proper sleep duration to benefit metabolic function, while older adults should pay more attention to whether their metabolism-related diseases have made an influence on sleep or other behaviors, which may, in turn causing more serious health problems. The results of the stratified analysis also showed that the cross-lagged path coefficients were more significant in adults with BMI > 25. One possible sociological explanation is that sleep patterns are less affected by other social factors in those full-bodied people, since sleep patterns could be greatly influenced by poverty and a fuller body usually equals more wealth and a higher social status among the older generation in China. It is worth noting that because HDL-C is positively correlated with BMI in our study population, the effect of sleep duration on HDL-C may be masked by weight-related factors in the participants with higher BMI.

### Strengths and Limitations

There are several limitations that need to be considered in our study. First, the subjective self-reported sleep duration obtained through the questionnaire might be biased, which could be caused by systematic over-reporting. Second, although several potential confounders were included in our analysis, there was a lack of physical performance, diet measures and menopause status, which could be related to sleep and blood lipid levels (44). Third, measures of insomnia, obstructive sleep apnea syndrome and sleep symptoms, such as snoring, which have been suggested to be associated with metabolic syndrome (45), were not investigated in the present study. Fourth, although the number of eligible subjects in the study was large enough, the proportions of participants who were successfully followed up and were able to be collected blood samples at each time point were about 70%, which might lead to selection bias common in longitudinal studies. Lastly, although path analysis permits the exploration directional effect variables have on each other over time, it simply assumed that the research variables are linearly correlated. In the present study, there was no obvious other relationship such as “V” or “U”-shape between variables and the proposed models showed a good fit to the data; however, there may be more subtle relationship between variables, and the shape of associations may vary among the population of different ages or races. Hence, further prospective and mechanistic studies are required to validate our results on timing relationships and causality.

In conclusion, this study aimed to clarify the temporal relationship between blood lipids and sleep duration in Chinese middle-aged and older adults by employing the two-wave cross-lagged design. Our research revealed that negative associations existed between HDL-C and sleep duration and the relationships were bidirectional across the general population. TG has a positive longitudinal association with sleep duration, and sleep duration is negatively associated with LDL-C and TC. The strength and direction of the relationship are inconsistent among different ages and BMI groups. The longitudinal association between HDL-C at baseline and sleep duration at follow-up was more pronounced in individuals with BMI >25 or age≥60 years, while the association in the opposite direction was only observed in the other groups. Similarly, the significant effect of TG on sleep duration was not observed in individuals with BMI ≤ 25 or age <60 years. Further mechanism studies are necessary for providing evidence for causality.

## Data Availability Statement

Publicly available datasets were analyzed in this study. This data can be found here: All data in CHARLS are maintained at Peking University and are accessible publicly. CHARLS data are available at http://charls.pku.edu.cn/pages/data/111/zh-cn.html.

## Ethics Statement

The studies involving human participants were reviewed and approved by the approval for CHARLS data was obtained from the Biomedical Ethics Review Committee of Peking University (approval number: IRB00001052-11015). Ethics approval for the use of CHARLS data was obtained from the University of Newcastle Human Research Ethics Committee (H-2015-0290). All participants signed informed consent at the time of participation. The patients/participants provided their written informed consent to participate in this study.

## Author Contributions

ZC and YM: conceptualization, methodology, data curation and analysis, and original draft preparation. YD, TM, and WL: validation, visualization, and critical revision of the manuscript. XZ and PY: investigation, data interpretation, and technical support. XZ: methodology and reviewing and editing. PY: administrative support and supervision. All authors contributed to the article and approved the submitted version.

## Funding

This work was founded by the National Natural Science Foundation of China (No. 82173628).

## Conflict of Interest

The authors declare that the research was conducted in the absence of any commercial or financial relationships that could be construed as a potential conflict of interest.

## Publisher's Note

All claims expressed in this article are solely those of the authors and do not necessarily represent those of their affiliated organizations, or those of the publisher, the editors and the reviewers. Any product that may be evaluated in this article, or claim that may be made by its manufacturer, is not guaranteed or endorsed by the publisher.

## Abbreviations

BMI: Body Mass Index
CAPIs: computer- assisted personal interviews
CESD-10: Center for Epidemiologic Studies Depression Scale
CFI: comparative fit index
CHARLS: China Health and Retirement Longitudinal Study
HDL-C: high-density lipoprotein cholesterol
LDL-C: low-density lipoprotein cholesterol
TC: total cholesterol
RMSEA: Root Mean Square Error of Approximation
SEM: Standard Error of Mean
SRMR: Standardized Root Mean Square Residual
TG: Triglycerides.

## References

[1] Magee CA Kritharides L Attia J Mcelduff P Banks E. Short and long sleep duration are associated with prevalent cardiovascular disease in Australian adults. J Sleep Res. (2012) 21:441–7. 10.1111/j.1365-2869.2011.00993.x 22211671

[2] Itani O Jike M Watanabe N Kaneita Y. Short sleep duration and health outcomes: a systematic review, meta-analysis, and meta-regression. Sleep Med. (2017) 32:246–56. 10.1016/j.sleep.2016.08.006 27743803

[3] Yan M Fu Z Qin T Wu NJ Lv YL Wei QY. Associations of sleep duration and prediabetes prevalence in a middle-aged and elderly Chinese population with regard to age and hypertension: the China Health and Retirement Longitudinal Study baseline survey. J Diabetes. (2018) 10:847–56. 10.1111/1753-0407.12662 29573578

[4] Gallicchio L Kalesan B. Sleep duration and mortality: a systematic review and meta-analysis. J Sleep Res. (2009) 18:148–58. 10.1111/j.1365-2869.2008.00732.x 19645960

[5] Liu TZ Xu C Rota M Cai H Zhang C Shi MJ. Sleep duration and risk of all-cause mortality: a flexible, non-linear, meta-regression of 40 prospective cohort studies. Sleep Med Rev. (2017) 32:28–36. 10.1016/j.smrv.2016.02.005 27067616

[6] Navar-Boggan AM Peterson ED D'Agostino RB Neely B Sniderman AD Pencina MJ. Hyperlipidemia in early adulthood increases long-term risk of coronary heart disease. Circulation. (2015) 131:451–8. 10.1161/CIRCULATIONAHA.114.012477 25623155 PMC4370230

[7] Gao NN Yu Y Zhang BC Yuan ZS Zhang HQ Song YF. Dyslipidemia in rural areas of North China: prevalence, characteristics, and predictive value. Lipids Health Dis. (2016) 15:154. 10.1186/s12944-016-0328-y 27619340 PMC5020547

[8] Yang W Xiao J Yang Z Ji LN Jia WP Weng JP. Serum lipids and lipoproteins in Chinese men and women. Circulation. (2012) 125:2212–21. 10.1161/CIRCULATIONAHA.111.065904 22492668

[9] Bjorvatn B Sagen Im Øyane N Waage S Fetveit A Pallesen S. The association between sleep duration, body mass index and metabolic measures in the Hordaland Health Study *.J Sleep Res*. (2007) 16:66–76. 10.1111/j.1365-2869.2007.00569.x 17309765

[10] Arora T Jiang CQ Thomas GN Lam KBH Zhang WS Cheng KK. Self-reported long total sleep duration is associated with metabolic syndrome: the Guangzhou Biobank Cohort Study. Diabetes Care. (2011) 34:2317–9. 10.2337/dc11-0647 21873559 PMC3177714

[11] Hall MH Muldoon MF Jennings JR Buysse DJ Flory JD Manuck SB. Self-reported sleep duration is associated with the metabolic syndrome in midlife adults. Sleep. (2008) 31:635–43. 10.1093/sleep/31.5.635 18517034 PMC2398755

[12] Kim JY Yadav D Ahn SV Koh SB Park JT Yoon J. A prospective study of total sleep duration and incident metabolic syndrome: the ARIRANG study. Sleep Med. (2015) 16:1511–5. 10.1016/j.sleep.2015.06.024 26611949

[13] Gangwisch JE Malaspina D Babiss LA Opler MG Posner K Shen S. Short sleep duration as a risk factor for hypercholesterolemia: analyses of the national longitudinal study of adolescent health. Sleep. (2010) 33:956–61. 10.1093/sleep/33.7.956 20614855 PMC2894437

[14] Yang L Yang H He M Pan A Li XL Min XW. Longer sleep duration and midday napping are associated with a higher risk of CHD incidence in middle-aged and older Chinese: the Dongfeng-Tongji Cohort Study. Sleep. (2016) 39:645–52. 10.5665/sleep.5544 26564127 PMC4763372

[15] Kinuhata S Hayashi T Sato KK Uehara S Oue K Endo G. Sleep duration and the risk of future lipid profile abnormalities in middle-aged men: the Kansai Healthcare Study. Sleep Med. (2014) 15:1379–85. 10.1016/j.sleep.2014.06.011 25220668

[16] Kaneita Y Uchiyama M Yoshiike N Ohida T. Associations of usual sleep duration with serum lipid and lipoprotein levels. Sleep. (2008) 31:645–52. 10.1093/sleep/31.5.645 18517035 PMC2398756

[17] Byrne DW Rolando LA Aliyu MH McGown PW Connor LR Awalt BM. Modifiable healthy lifestyle behaviors: 10-year health outcomes from a health promotion program. Am J Prev Med. (2016) 51:1027–37. 10.1016/j.amepre.2016.09.012 27866595

[18] Lu Y Wang P Zhou T Lu JP Spatz ES Nasir K. Comparison of prevalence, awareness, treatment, and control of cardiovascular risk factors in China and the United States. J Am Heart Assoc. (2018) 7:e007462. 10.1161/JAHA.117.007462 29374046 PMC5850247

[19] Cappuccio FP Miller MA. Sleep and mortality: cause, consequence, or symptom? Sleep Med. (2013) 14:587–8. 10.1016/j.sleep.2013.04.001 23684937

[20] Grandner MA Hale L Moore M Patel NP. Mortality associated with short sleep duration: the evidence, the possible mechanisms, and the future. Sleep Med Rev. (2010) 14:191–203. 10.1016/j.smrv.2009.07.006 19932976 PMC2856739

[21] Zhao Y Hu Y Smith JP Strauss J Yang GH. Cohort profile: the China Health and Retirement Longitudinal Study (CHARLS). Int J Epidemiol. (2014) 43:61–8. 10.1093/ije/dys203 23243115 PMC3937970

[22] Cheng ST Chan ACM. The Center for Epidemiologic Studies Depression Scale in older Chinese: thresholds for long and short forms. Int J Geriatr Psych. (2005) 20:465–70. 10.1002/gps.1314 15852439

[23] Alberti KGMM Eckel RH Grundy SM Zimmet PZ Cleeman JI Donato KA. Harmonizing the metabolic syndrome: a joint interim statement of the International Diabetes Federation Task Force on Epidemiology and Prevention; National Heart, Lung, and Blood Institute; American Heart Association; World Heart Federation; International Atherosclerosis Society; and International Association for the Study of Obesity. Circulation. (2009) 120:1640–5. 10.1161/CIRCULATIONAHA.109.192644 19805654

[24] Zhao YH, Strauss, J, Yang, GH, Giles, J, Hu, PF, Hu, YS, . China Health Retirement Longitudinal Study, 2011-2012 National Baseline Users' Guide. National School of Development, Peking University (2013). Available online at: http://charls.pku.edu.cn/wenjian/jixiandiaochashujuyonghushiyongshouce2013-04-07.pdf

[25] Kenny DA. Cross-lagged panel correlation: a test for spuriousness. Psychol Bull. (1975) 82:887–903. 10.1037/0033-2909.82.6.8877108742

[26] Chen W Li S Fernandez C Sun DJY Lai CC Zhang T. Temporal relationship between elevated blood pressure and arterial stiffening among middle-aged black and white adults: the bogalusa heart study. Am J Epidemiol. (2016) 183:599–608. 10.1093/aje/kwv274 26960706 PMC4801137

[27] Yan Y Li S Liu Y Bazzano L He J Mi J. Temporal relationship between inflammation and insulin resistance and their joint effect on hyperglycemia: the Bogalusa Heart Study. Cardiovasc Diabetol. (2019) 18:109. 10.1186/s12933-019-0913-2 31443647 PMC6706925

[28] Kline RB. Pinciples and Practice of Structural Equation Modeling. 4th ed. New York, NY, Guilford (2016).

[29] Bentler PM. Comparative fit indexes in structural models. Psychol Bull. (1990) 107:238–46. 10.1037/0033-2909.107.2.238 2320703

[30] Hu LT Bentler PM. Cutoff criteria for fit indexes in covariance structure analysis: conventional criteria versus new alternatives. Struct Equ Model. (1999) 6:1–55. 10.1080/10705519909540118

[31] Rosseel Y. lavaan: An R package for structural equation modeling. J Stat Softw. (2012) 48:1–36. 10.18637/jss.v048.i0225601849

[32] Li X Lin L Lv L Pang XY Du SS Zhang W. U-shaped relationships between sleep duration and metabolic syndrome and metabolic syndrome components in males: a prospective cohort study. Sleep Med. (2015) 16:949–54. 10.1016/j.sleep.2015.03.024 26116460

[33] Grandner MA Schopfer EA Sands-Lincoln M Jackson N Malhotra A. Relationship between sleep duration and body mass index depends on age. Obesity. (2015) 23:2491–8. 10.1002/oby.21247 26727118 PMC4700549

[34] Petrov MER Kim Y Lauderdale D Lewis CE Reis JP Carnethon MR. Longitudinal associations between objective sleep and lipids: the CARDIA study. Sleep. (2013) 36:1587–95. 10.5665/sleep.3104 24179290 PMC3792374

[35] Kruisbrink M Robertson W Ji C Miller MA Geleijnse JM Cappuccio FP. Association of sleep duration and quality with blood lipids: a systematic review and meta-analysis of prospective studies. BMJ Open. (2017) 7:e018585. 10.1136/bmjopen-2017-018585 29247105 PMC5735405

[36] Grandner MA. Sleep, health, and society. Sleep Med Clin. (2017) 12:1–22. 10.1016/j.jsmc.2016.10.012 28159089 PMC6203594

[37] Budoff M. Triglycerides and triglyceride-rich lipoproteins in the causal pathway of cardiovascular disease. Am J Cardiol. (2016) 118:138–45. 10.1016/j.amjcard.2016.04.004 27184174

[38] Araghi MH Thomas GN Taheri S. The potential impact of sleep duration on lipid biomarkers of cardiovascular disease. Clin Lipidol. (2012) 7:443–53. 10.2217/clp.12.43

[39] Theorell-Haglow J Berglund L Berne C Lindberg E. Both habitual short sleepers and long sleepers are at greater risk of obesity: a population-based 10-year follow-up in women. Sleep Med. (2014) 15:1204–11. 10.1016/j.sleep.2014.02.014 25113417

[40] Firmann M Mayor V Vidal PM Bochud M Pecoud A Hayoz D. The CoLaus study: a population-based study to investigate the epidemiology and genetic determinants of cardiovascular risk factors and metabolic syndrome. BMC Cardiovasc Disord. (2008) 8:6. 10.1186/1471-2261-8-6 18366642 PMC2311269

[41] Saklayen MG. The global epidemic of the metabolic syndrome. Curr Hypertens Rep. (2018) 20:12. 10.1007/s11906-018-0812-z 29480368 PMC5866840

[42] Ohayon MM Carskadon MA Guilleminault C Vitiello MV. Meta-analysis of quantitative sleep parameters from childhood to old age in healthy individuals: developing normative sleep values across the human lifespan. Sleep. (2004) 27:1255–73. 10.1093/sleep/27.7.1255 15586779

[43] Buxton OM Cain SW O'Connor SP Porter JH Duffy JF Wang W. Adverse metabolic consequences in humans of prolonged sleep restriction combined with circadian disruption. Sci Transl Med. (2012) 4:129–43. 10.1126/scitranslmed.3003200 22496545 PMC3678519

[44] National Health Service. High *cholesterol; c2019*. Available online at: http://www.nhs.uk/conditions/Cholesterol/Pages/Introduction.aspx (accessed April 10, 2020).

[45] Troxel WM Buysse DJ Matthews KA Kip KE Strollo PJ Hall M. Sleep symptoms predict the development of the metabolic syndrome. Sleep. (2010) 33:1633–40. 10.1093/sleep/33.12.1633 21120125 PMC2982733

